# One-Pot Synthesis of 2-Phenylimidazo[1,2-α]pyridines from Acetophenone, [Bmim]Br_3_ and 2-Aminopyridine under Solvent-Free Conditions

**DOI:** 10.3390/molecules171113368

**Published:** 2012-11-09

**Authors:** Zhang-Gao Le, Zong-Bo Xie, Jian-Ping Xu

**Affiliations:** Department of Applied Chemistry, East China Institute of Technology, Fuzhou 344000, China

**Keywords:** imidazo[1,2-α]pyridine, aromatics, 2-aminopyridine, [Bmim]Br_3_

## Abstract

One-pot synthesis of 2-phenylimidazo[1,2-α]pyridines from acetophenone, [Bmim]Br_3_ and 2-aminopyridine under solvent-free conditions in the presence of Na_2_CO_3_, gave the corresponding 2-phenylimidazo[1,2-α]pyridines in excellent yields ranging from 72% to 89%.

## 1. Introduction

Imidazo[1,2-α]pyridines are of interest because of the occurrence of their saturated and partially saturated derivatives in a variety of biologically active compounds, which have been shown to possess a broad range of useful pharmacological properties [1], including anti-inflammatory [2,3], antiprotozoal [4], antiviral [5,6,7], antiulcer [8,9], antibacterial [10,11], antifungal [12], antiprotozoal [4,13], antiherpes [14,15] and treatment of hepatitis C [16], and HIV [17], *etc.* They are also versatile intermediates for synthetic transformations [18,19].

**Scheme 1:**
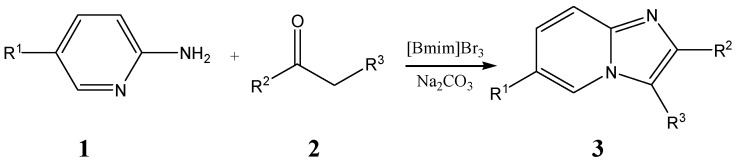
Synthesis of 2-phenylimidazo[1,2-α]pyridines from acetophenone, [Bmim]Br_3_ and 2-aminopyridine.

Various methods have been reported for the synthesis of substituted imidazo[1,2-a]pyridines. The most important approaches embrace [20]: (1) condensation of 2-aminopyridine with α-halocarbonyl compounds [21,22,23,24], (2) one-pot condensations of isocyanide, aldehydes, and 2-aminopyridines [25,26,27], and (3) three-component reactions of 2-aminopyridines, aldehydes, and alkynes [28,29,30]. Although new methods are being published continuously, the synthesis of imidazo[1,2-α]pyridines using 2-aminopyridines with α-haloketones and α-haloaldehydes is still the most popular [31,32]. There are however two intrinsic limitations to this methodology, namely, the small variety of commercially available α-halocarbonyl compounds and their lachrymatory properties. Generally, α-bromoketones can be obtained by reaction of ketones with various reagents such as bromine [33], copper (ІІ) bromide [34], dioxane dibromide [35], polymer-supported pyridinium bromide perbromide [36], and N-bromosuccinimide [37], *etc.* All these methods involve the use of expensive reagents and harmful organic solvents, long reaction times, high temperatures and sometimes only give poor yields, so we report here a simple, efficient method for a mild one-pot synthesis of 2-phenylimidazo[1,2-α]pyridines from acetophenone, [Bmim]Br_3_ and 2-aminopyridine (Scheme 1).

## 2. Results and Discussion

Firstly, we found that the reaction of acetophenone, 1-butyl-3-methylimidazolium tribromide ([Bmim]Br_3_) and 2-aminopyridine proceeds smoothly in the presence of Na_2_CO_3_ at room temperature to form 2-phenylimidazo[1,2-α]pyridine in 82% yield (Table 1, entry 1). In a similar fashion, a variety of acetophenones reacted smoothly with [Bmim]Br_3_ and 2-aminopyridines under these reaction conditions to give the corresponding substituted 2-phenylimidazo[1,2-α]pyridines. The results are summarized in Table 1.

**Table 1:** Synthesis of 2-phenylimidazo[1,2-α]pyridines from acetophenone, [Bmim]Br_3 _and 2-aminopyridine under solvent-free conditions ^a^.

Entry	R^1^	R^2^	R^3^	Product	Yield ^b^ (%)	Mp (°C)	Lit. mp (°C)
1	H	C_6_H_5_	H	**3a**	82	136–137	136–137 [24]
2	H	*p*-MeC_6_H_4_	H	**3b**	77	144–145	144–145 [38]
3	H	*p*-MeOC_6_H_4_	H	**3c**	75	134–135	135–136 [24]
4	H	*p*-FC_6_H_4_	H	**3d**	89	164–165	165–166 [39]
5	H	*p*-ClC_6_H_4_	H	**3e**	86	201–202	201 [40]
6	H	2,4-Cl_2_C_6_H_3_	H	**3f**	72	182	181–182 [24]
7	H	*p*-BrC_6_H_4_	H	**3g**	87	216	215–216 [41]
8	H	C_6_H_5_	Me	**3h**	77	160–161	159–161 [24]
9	Cl	C_6_H_5_	H	**3i**	81	205–206	204–206 [24]
10	Cl	*p*-MeC_6_H_4_	H	**3j**	80	224–225	223–224 [38]
11	Cl	*p*-MeOC_6_H_4_	H	**3k**	75	236–237	234–236 [24]
12	Cl	*p*-FC_6_H_4_	H	**3l**	78	191–192	191–192 [38]
13	Cl	*p*-ClC_6_H_4_	H	**3m**	77	206–207	205–207 [24]
14	Me	C_6_H_5_	H	**3n**	76	172–173	171–173 [24]
15	Me	*p*-MeOC_6_H_4_	H	**3o**	84	181–182	179–181 [24]
16	Me	*p*-ClC_6_H_4_	H	**3p**	81	140–141	239–240 [24]

^a^ All reaction were run with acetophenone (2 mmol), [Bmim]Br_3_ (2 mmol), Na_2_CO_3_ (1.1 mmol) and 2-aminopyridine (2.4 mmol) at r. t. ^b^ Isolated yield.

All the products gave satisfactory m.p., and ^1^H-NMR spectra which were consistent with the literature data. As can be seen from Table 1, the reaction is general and applicable to acetophenones bearing various groups such as methyl, methoxy, fluoro, chloro, bromo, *etc*. (Table 1, entries 1–7). On the basis of these results, we extended our method to propiophenone. We found that this synthesis can also be performed in high yield (Table 1, entry 8). The experimental procedure is very simple and convenient. All of the tested reactions could complete within 40 min.

In order to explore the generality of the method, we conducted experiments with 2-amino-5-chloropyridine, 2-amino-5-methylpyridine and a variety of acetophenones containing different groups, such as methyl, methoxy, fluoro, chloro functions, which were also effective and gave the corresponding phenylimidazo[1,2-α]pyridine derivatives (Table 1, entries 9–16).

The present method has many obvious advantages compared to those reported in the literature, including higher yields, shorter reaction times, and solvent free conditions, thus being environmentally more benign. For example, the reactions of acetophenone with 2-aminopyridine by a recently reported method [20] gave 2-phenylimidazo[1,2-α]pyridine in 55% yield after ten hours at 110 °C, but using the present method, the same reaction was completed smoothly and gave the product with isolated yields of 82%. Using Xie’s reported method [39], preparation of 2-(4-fluorophenyl)imidazo[1,2-α]pyridine and 2-(4-chlorophenyl)imidazo[1,2-α]pyridine by cyclocondensation of 4-fluoroacetophenone, 4-chloro- acetophenone with [hydroxy(tosyloxy)iodo]benzene and 2-aminopyridine in the ionic liquid (BPyBF4) was successful and gave yields 85% and 74%. The same reaction was completed smoothly with the present method and gave higher yields (89%, 86%).

## 3. Experimental

### 3.1. General

Melting points were determined on a digital melting point apparatus and are not corrected. Nuclear magnetic resonance spectra were recorded on a Bruker AVANCE DMX 400 spectrometer in CDCl_3_ using TMS as an internal standard. The ionic liquid [Bmim]Br_3_ was synthesized according to a reported procedure [42]. The other materials are commercially available and were used without further purification.

### 3.2. General Procedure for the Synthesis of 2-phenylimidazo[1,2-α]pyridines

[Bmim]Br_3_ (2 mmol) was very slowly added (1 drop per 5 s) to acetophenone (2 mmol) with continuous stirring for 5 min at room temperature (30 °C). Then Na_2_CO_3_ (1.1 mmol) and 2-aminopyridine (2.4 mmol) were added, and the mixture was stirred at room temperature for 40 min. After the reaction completion, the reaction mixture was extracted with Et_2_O, the ethereal layer was concentrated by rotary evaporator, and the crude product was purified by the preparative thin-layer chromatography on silica gel using a mixture of petroleum ether and EtOAc as developer to give the corresponding pure products of 2-phenylimidazo[1,2-α]pyridines.

### 3.3. Spectroscopic Data

*2-Phenylimidazo[1,2-α]pyridine* (**3a**). ^1^H-NMR: δ = 6.77–6.79 (t, *J* = 6.7 Hz, 1 H), 7.16–7.19 (m, 1 H), 7.32–7.35 (t, 1 H), 7.43–7.46 (m, 2 H), 7.63–7.65 (d, *J* = 9.1 Hz, 1 H), 7.86 (s, 1 H), 7.95–7.97 (t, 2 H), 8.12 (d, *J* = 6.8 Hz, 1 H).

*2-(4-Methylphenyl)imidazo[1,2-α]pyridine* (**3b**). ^1^H-NMR: δ = 2.39 (s, 3 H), 6.77–6.79 (t, *J* = 6.7 Hz, 1 H), 7.16–7.18 (t, *J* = 7.9 Hz, 1 H), 7.24–7.26 (d, *J* = 8.2 Hz, 2 H), 7.64–7.65 (d, *J* = 9.1 Hz, 1 H), 7.82 (s, 1 H), 7.85–7.87 (d, *J* = 8.1 Hz, 2 H), 8.11 (d, *J* = 6.7 Hz, 1 H).

*2-(4-Methoxyphenyl)imidazo[1,2-α]pyridine* (**3c**). ^1^H-NMR: δ = 3.87 (s, 3 H), 6.74–6.78 (t, *J* = 6.8 Hz, 1 H), 6.97–6.99 (d, *J* = 6.8 Hz, 2 H), 7.14–7.17 (t, *J* = 7.9 Hz, 1 H), 7.61 (d, *J* = 9.1 Hz, 1 H), 7.78 (s, 1 H), 7.89 (d, *J* = 6.8 Hz, 2 H), 8.1 (d, *J* = 6.8 Hz, 1 H).

*2-(4-Fluorophenyl)imidazo[1,2-α]pyridine* (**3d**). ^1^H-NMR: δ = 6.78–6.81 (t, *J* = 6.8 Hz, 1 H), 7.11–7.18 (m, 3 H), 7.62–7.64 (d, *J* = 9.1 Hz, 1 H), 7.82 (s, 1 H), 7.91–7.95 (m, 2 H), 8.12 (d, *J* = 6.8 Hz, 1 H).

*2-(4-Chlorophenyl)imidazo[1,2-α]pyridine* (**3e**). ^1^H-NMR: δ = 6.78–6.81 (t, *J* = 6.7 Hz, 1 H), 7.18–7.21 (m, 1 H), 7.39–7.42 (m, 2 H), 7.61–7.64 (d, *J* = 9.1 Hz, 1 H), 7.84 (s, 1 H), 7.88–7.90 (m, 2 H), 8.12 (d, *J* = 6.8 Hz, 1 H).

*2-(2,4-Dichlorophenyl)imidazo[1,2-α]pyridine* (**3f**).^1^H-NMR: δ = 6.80 (t, *J* *=* 6.8, 1H), 7.17–7.23 (m, 1 H), 7.36 (dd, *J =* 2.0, 2.0, 1 H), 7.49-7.50 (m, 1 H), 7.62 (d, *J* *=* 9.1, 1 H), 8.14 (d, *J =* 6.8, 1 H), 8.26–8.28 (m, 2H).

*2-(4-Bromophenyl)imidazo[1,2-α]pyridine* (**3g**). ^1^H-NMR: δ = 6.78–6.82 (t, *J* = 6.7 Hz, 1 H), 7.18–7.21 (m, 1 H), 7.55–7.57 (m, 2 H), 7.62–7.64 (d, *J* = 9.1 Hz, 1 H), 7.81 (s, 1 H), 7.83–7.85 (m, 2 H), 8.11 (d, *J* = 6.8 Hz, 1 H).

*3-Methyl-2-phenylimidazo[1,2-α]pyridine* (**3h**). ^1^H-NMR: δ = 2.64 (s, 3 H), 6.85–6.88 (t, *J* = 6.8 Hz, 1 H), 7.16–7.19 (t, *J* = 7.9 Hz, 1 H), 7.34–7.37 (m, 1 H), 7.45–7.49 (m, 2 H), 6.64–6.67 (d, *J* = 9.1 Hz, 1 H), 7.79–7.82 (m, 2 H), 7.91 (d, *J* = 6.8 Hz, 1 H).

*6-Chloro-2-phenylimidazo[1,2-α]pyridine* (**3i**). ^1^H-NMR: δ = 7.15 (d, *J =* 9.4, 1H), 7.34 (d, *J =* 7.2, 1 H), 7.38-7.51 (m, 2 H), 7.57 (d, *J =* 9.1, 1 H), 7.82 (s, 1 H), 7.95 (d, *J =* 7.3, 2 H), 8.16 (s, 1 H).

*6-Chloro-2-(4-methylphenyl)imidazo[1,2-α]pyridine* (**3j**). ^1^H-NMR: δ = 2.39 (s, 3 H), 7.1 (d, *J* = 9.6 Hz, 1 H), 7.23 (d, *J =* 7.8, 2 H), 7.54 (d, *J =* 9.6, 1 H), 7.74 (s, 1 H), 7.80 (d, *J* = 8.1 Hz, 2 H), 8.10 (s, 1 H).

*6-Chloro-2-(4-methoxyphenyl)imidazo[1,2-α]pyridine* (**3k**). ^1^H-NMR: δ = 3.87 (s, 3 H), 7.00 (dd, *J =* 2.0, 2.0, 2 H), 7.13 (dd, *J =* 2.0, 2.0, 1 H), 7.56 (d, *J =* 9.54, 1 H), 7.76 (s, 1 H), 7.88 (dd, *J =* 2.0, 2.0, 2 H), 8.15-8.16 (m, 1 H).

*6-Chloro-2-(4-Fluorophenyl)imidazo[1,2-α]*pyridine (**3l**). ^1^H-NMR: δ = 6.91–7.15 (m, 3 H), 7.53 (d, *J* = 9.6 Hz, 1 H), 7.71 (s, 1 H), 7.85–7.91 (m, 2 H), 8.10 (dd, *J* = 2.1, 0.7 Hz, 1 H).

*6-Chloro-2-(4-chlorophenyl)imidazo[1,2-α]pyridine* (**3m**). ^1^H-NMR: δ = 7.15 (dd, *J =* 2.0, 2.0, 1 H), 7.40–7.42 (m, 2 H), 7.55 (d, *J =* 9.6, 1 H), 7.76 (s, 1 H), 7.85 (dd, *J =* 2.0, 2.0, 2 H), 8.13–8.14 (m, 1 H).

*6-Methyl-2-phenylimidazo[1,2-α]pyridine* (**3n**). ^1^H-NMR: δ = 2.30 (s, 3 H), 7.00 (d, *J =* 8.8, 1 H), 7.33 (d, *J =* 6.4, 1 H), 7.44 (t, *J =* 6.6, 2 H), 7.53 (d, *J =* 9.1, 1 H), 7.74 (s, 1 H), 7.83 (s, 1 H), 7.97 (d, *J =* 7.1, 2 H).

*6-Methyl-2-(4-methoxyphenyl)imidazo[1,2-α]pyridine* (**3o**). ^1^H-NMR: δ = 2.33 (s, 3 H), 3.86 (s, 3 H), 6.97 (dd, *J =* 2.1, 2.1, 1 H), 7.01 (d, *J =* 1.6, 2 H), 7.51 (d, *J =* 9.5, 1 H), 7.68 (s, 1 H), 7.87 (d, *J =* 2.1, 1 H), 7.89 (d, *J =* 2.1, 2 H).

*6-Methyl-2-(4-chlorophenyl)imidazo[1,2-α]pyridine* (**3p**). ^1^H-NMR: δ = 2.34 (s, 3 H), 7.05 (d, *J* = 9.3, 1 H), 7.39 (d, *J* = 8.5, 2 H), 7.54 (t, *J* = 6.0, 1 H), 7.76 (s, 1 H), 7.88 (s, 1 H), 7.90 (d, *J* = 2.8, 2 H). 

## 4. Conclusions

In conclusion, we have demonstrated that the synthesis of 2-Phenylimidazo[1,2-α]pyridines from acetophenone, [Bmim]Br_3 _and 2-aminopyridine under solvent-free conditions in the presence of Na_2_CO_3_, which provides a simple efficient method for the synthesis of 2-phenylimidazo[1,2-α]pyridines. The present method has many obvious advantages compared to those reported in the literature, including avoiding to using toxic solvent or catalyst, being environmentally more benign, the simplicity of the methodology, the ease of product isolation, and the higher yield.
